# Direct Writing Supercapacitors Using a Carbon Nanotube/Ag Nanoparticle-Based Ink on Cellulose Acetate Membrane Paper

**DOI:** 10.3390/polym11060973

**Published:** 2019-06-03

**Authors:** Xipeng Guan, Lin Cao, Qin Huang, Debin Kong, Peng Zhang, Huaijun Lin, Wei Li, Zhidan Lin, Hong Yuan

**Affiliations:** 1 MOE Key Laboratory of Disaster Forecast and Control in Engineering, School of Mechanics and Construction Engineering, Jinan University, Guangzhou 510632, China; guanxipeng@jnu.edu.cn; 2 Institute of Advances Wear & Corrosion Resistant and Functional Materials, Jinan University, Guangzhou 510632, China; linc19993@163.com (L.C.); hq2502@126.com (Q.H.); 13580457530@163.com (D.K.); tzhangpeng@jnu.edu.cn (P.Z.); hjlin@jnu.edu.cn (H.L.); liweijnu@126.com (W.L.)

**Keywords:** Ag, CNT, flexible supercapacitor electrode, polymer conductive film, cellulose acetate membrane

## Abstract

In this work, we present a cellulose acetate membrane flexible supercapacitor prepared through a direct writing method. A carbon nanotube (CNT) and silver (Ag) nanoparticle were prepared into ink for direct writing. The composite electrode displayed excellent electrochemical and mechanical electrochemical performance. Furthermore, the CNT-Ag displayed the highest areal capacity of 72.8 F/cm^3^. The assembled device delivered a high areal capacity (17.68 F/cm^3^) at a current density of 0.5 mA/cm^2^, a high areal energy (9.08–5.87 mWh/cm^3^) at a power density of 1.18–0.22 W/cm^3^, and showed no significant decrease in performance with a bending angle of 180°. The as-fabricated CNT/Ag electrodes exhibited good long-term cycling stability after 1000 time cycles with 75.92% capacitance retention. The direct writing was a simple, cost-effective, fast, and non-contact deposition method. This method has been used in current printed electronic devices and has potential applications in energy storage.

## 1. Introduction

With the development of the social economy, human beings are paying more and more attention to green energy and the ecological environment [1,2,3,4]. As a new type of energy storage device, the supercapacitor is generally environmentally friendly and has an irreplaceable superiority [5,6]. The two most important points in the design and fabrication of flexible supercapacitors are electrode materials and structure. In the past few years, the number of scientific articles in the fields of laser, direct writing, and printing has increased significantly [7,8,9]. The direct writing exhibits advantages such as low temperature, being environmentally friendly, shorter reaction times, energy saving, and excellent control over experimental parameters. In addition, the direct writing method allows the active material to undergo catalyst-free growth on insulating substrates.

Carbon-based materials have become attractive nanomaterials in various applications due to their unique properties [10]. A carbon-based nanotube (CNT) has a unique hollow structure, a high specific surface area, mechanical flexibility, a high stability, and a low cost, and it is suitable for electrolyte ion migration pores. Its use as an electrode material can significantly improve the power characteristics and frequency response characteristics of supercapacitors. Additionally, supercapacitors using carbon material-based materials mainly rely on the electrical double layer capacitor (EDLC), exhibiting fast charge-discharge times and surface-area-dependent capacities [11,12,13]. At present, many studies are devoted to the study of conductive patterns in carbon nanotube printing [14,15,16]. However, the printed carbon nanotubes have an overly high resistance on flexible substrates and cannot be widely used in optoelectronic devices [17,18].

Both Ag nanowires and sintered Ag nanoparticles have a high conductivity [19,20,21,22]. However, nanoparticles are easier to print without clogging the nozzle. The mixing of Ag nanoparticles with graphene can significantly reduce the sheet resistance [23]. Therefore, the mixing of Ag nanoparticles with carbon nanotubes can also reduce the sheet resistance without changing the mechanical flexibility. Combining metal nanoparticles with CNTs can combine the excellent properties of these two types of nanomaterials, and the obtained nanoassembled materials have great application potential in the fields of optics, electricity, and electrocatalysis. The micro/nanostructured metals and metal oxides, such as Ag, Au, Ru_x_O_y_, Mn_x_O_y_, and Co_x_O_y_, as well as their hybrids, can facilitate electron charge transfer induced by electrosorption, redox reactions, and intercalation processes at the interfaces, thereby remarkably improving the energy densities [24,25,26,27,28,29].

The cellulose acetate membrane paper is usually composed of cellulose fibers arranged in *N*-dimensional form, and the surface of the cellulose acetate membrane paper is not only very rough but also has a highly porous structure as compared with a conventional flexible substrate. In particular, we can make full use of the porous structure of the cellulose film [30,31,32] to prepare high-performance flexible electrodes.

In this work, we report a low-temperature, fast, controlled, and straightforward approach for the fabrication of CNT/Ag electrodes on a flexible cellulose acetate membrane by the direct writing method. In the new energy system of CNT/Ag, CNT contribution of specific capacitance serves as carbon support and Ag promotion of electrical conductivity as a conducting agent, while the cellulose acetate membrane provides support and a porous structure as a flexible substrate. Furthermore, the capacitive behaviors of the CNT/Ag electrode were tested mainly including cyclic voltammetry (CV), galvanostatic charge-discharge (GCD), and Nyquist, as well as the derivative specific capacitance. This work shows the potential application of flexible direct writing electrode materials for the operation of various flexible devices in the near future.

## 2. Materials and Methods

### 2.1. Materials

All the chemicals used in this study were of analytical reagent grade. H_2_SO_4_ and Na_2_SO_4_ were obtained from Guangzhou Chemical Reagent Factory (Guangzhou, China). Poly(vinyl alcohol) (PVA) was purchased from Shanghai Macklin Biochemical Co., Ltd. (Shanghai, China). Sodium dodecylbenzene sulfonate (SDBS) was provided by the Tianjin Damao Chemical Reagent Factory (Tianjin, China). Cellulose acetate membrane was provided by Tianjin Jinteng Experimental Equipment Co., Ltd. (Tianjin, China) CNT was purchased from Korea Kumho Corporation (Seoul, Korea). Ag nanoparticles (Ag) were obtained from the Shanghai Chaowei Nanotechnology Co., Ltd. (Shanghai, China).

### 2.2. Ag and CNT Ink Production

To prepare CNT ink, 0.20 g of SDBS was dissolved in 100 mL deionised water, after which 0.15 g of CNT was slowly added to the solution, and this was followed by ultrasonication for 0.5 h (ultrasonic power of 100 W, ultrasonic frequency of 80 kHz). For the Ag ink production, the 0.04, 0.12, and 0.20 g Ag nanoparticles were dissolved with 0.20 g SDBS in 100 mL deionised water, then added into a glass bottle, and the next procedure was similar to the preparation of the CNT ink.

### 2.3. Ag and MWCNT Electrode

**Figure 1:**
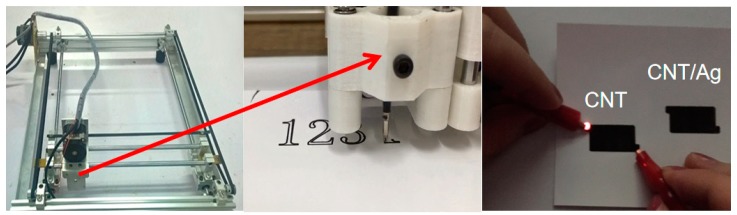
Overview of the fabrication process for direct-writing: design using a personal computer and direct-writing using a Plotter and a photo of patterned electrodes.

The process for rapid direct-write of the composite electrode consisted of three steps, as illustrated in Figure 1. During the entire direct-writing process, the cellulose acetate membrane paper was fixed on the experimental bench. First, CNT and Ag ink were printed onto the cellulose acetate membrane paper with a ballpoint pen core with an inner diameter of 1 mm. By using the venous infusion apparatus to provide liquid pressure and control, the writing speed was 500 mm/s by using a digital plotter. With a vacuum oven temperature of 60 °C, the ink was fully dried.

### 2.4. Characterization

Morphological observations were carried out using a field-emission scanning electron microscope (FE-SEM, Zeiss, Oberkochen, Germany). The resistance was tested using a KDB-1 digital four-probe resistance tester (Guangzhou Kunde Technology Companies (Guangzhou, China)). Galvanostatic charge-discharge (GCD), cyclic voltammetry (CV), and electrochemical impedance spectroscopy (EIS) were performed on the flexible electrode using a Princeton (PARSTAT 4000) electrochemical station. The capacitances (*C* in F·cm^−2^) of each device at different current densities were calculated from the discharge curves obtained from GCD tests using the following formula:

*C* = 2*I*Δt/*S*Δ*U*, (1)

where *I* is the applied discharge current (A), Δt is the discharge time (seconds), and Δ*U* (*V*) is the discharge voltage after the iR drop (ohmic voltage) is removed. *S* (cm^2^) is the volume of the active materials of all electrodes. Here, the surface areas of the active materials are 1 cm^2^.

The volumetric energy density and power density can provide more reliable performance metrics for porous, nanomaterial-based, thin-film devices compared to gravimetric capacitance. As a result, the volumetric energy density (Wh·cm^−2^) of each device was calculated using the following formula [33,34]:

*E* = 0.5*C*ΔU^2^/3.6, (2)

The volumetric power density (W·cm^−2^) of the device was calculated from the following formula:

*P* = 3600*E*/Δ*t*, (3)

We investigated the electrochemical properties of all electrodes on a three-electrode system using a Pt plate as the counter electrode and a saturated calomel reference electrode (SCE) in 1 M Na_2_SO_4_ aqueous electrolyte solution.

## 3. Results and Discussion

The fabrication steps of the electrodes and direct-write devices are illustrated in Figure 1. A personal computer was used to design the pattern of the electrodes, which were direct-written using a purchased plotter. The ink concentration was the most important factor for direct-writing, because a concentration that was too high clogged the top of the pen, and a concentration that was too low decreased the active substance content. Therefore, 5 wt % CNT and 1–5 wt % Ag were chosen for the printed ink.

**Figure 2:**
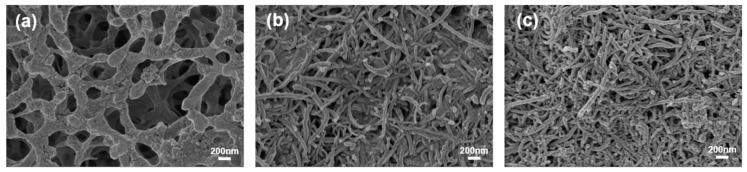
SEM images of (**a**) cellulose acetate membrane paper; (**b**) carbon nanotube (CNT); (**c**) CNT/Ag-5.

To further prepare the paper-based capacitor from a cellulose acetate membrane, we prepared a CNT and CNT/Ag ink for direct writing and prepared a paper-based capacitor with a cellulose acetate membrane by superposing the direct writing of CNT and Ag. As shown in Figure 2a, cellulose acetate membrane paper is usually composed of cellulose fibres arranged in an N-dimensional hierarchy, and the surface of the cellulose acetate membrane paper was not only very rough but also had a highly porous structure, in comparison to ordinary flexible substrates. We could also make full use of the porous structure of cellulose acetate membrane paper; when soaked, the active materials in the porous structure increased the contact area of the active material with the electrolyte. It could be used to improve the performance of electrochemical energy storage devices such as supercapacitors. The SEM image in Figure 2b shows that the CNT electrodes were composed of CNT with a diameter of about 50 nm. Then, different wt % Ag values were used to dope the CNT ink, and Ag particles were uniformly dispersed between carbon nanotubes (Figure 2c).

**Figure 3:**
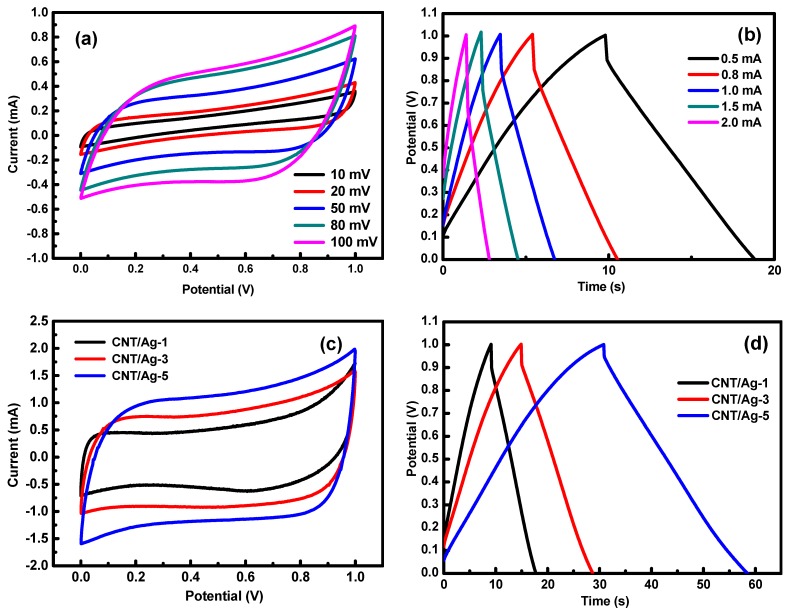

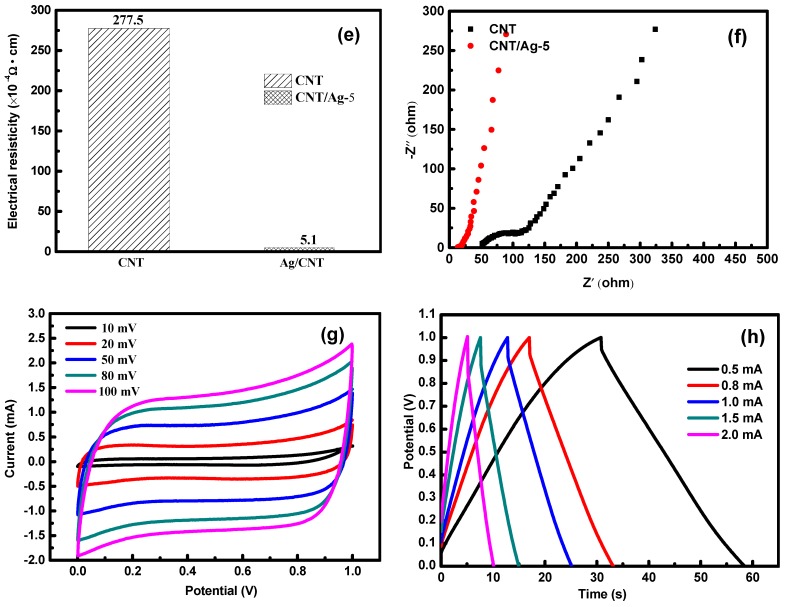
(**a**) Cyclic voltammetry (CVs) of the CNT electrodes with scan rate (from 10.0 to 100.0 mV/s). (**b**) Galvanostatic charge-discharge (GCD) of the CNT electrodes with 0.5 to 2.0 mA/cm^2^. (**c**) CV of CNT/Ag electrodes with different Ag. (**d**) GCD of CNT/Ag electrodes with different Ag. (**e**) Resistivity of the electrodes. (**f**) Nyquist plot of the electrodes. (**g**) CV of the CNT/Ag-5 electrodes with scan rate (from 10.0 to 100.0 mV/s). (**h**) GCD of the CNT/Ag-5 electrodes with 0.5 to 2.0 mA/cm^2^.

The CV, GCD, and EIS measurements were conducted in a three-electrode configuration in 1 M Na_2_SO_4_ aqueous solution. The results about the electrochemical performance of the CNT electrode before and after the doping of Ag nanoparticles are shown in Figure 3a–h. As expected, in Figure 3a, the rectangular CV curves of the electrode showed an ideal capacitive behavior. The GCD performance of the CNT electrode was further tested in Figure 3b. The volumetric capacitance for the CNT electrode was 25.3 F/cm^3^ at a discharge current density of 0.5 mA/cm^2^.

Moreover, the electrodes doping Ag exhibited a larger volume specific capacitance than CNT electrodes under the same voltage sweep speed (Figure 3a,c). The GCD curves of the CNT/Ag electrodes with different Ag doping doses at a current density of 0.5 mA/cm^2^ are shown in Figure 3d. It can be observed that as the Ag doping dose increased from 1 to 5 wt %, the charge and discharge time of CNT/Ag electrodes increased, and the iR drop decreased. This appearance indicates an increase in the capacitance of the CNT electrode after Ag doping. The volumetric capacitances for the 1, 3, and 5 wt % Ag doping CNT/Ag electrode were 25.7, 37.4, and 72.8 F/cm^3^, respectively, at a current density of 0.5 mA/cm^2^, which were much larger than the value of the CNT electrode (25.3 F/cm^3^). The high volumetric capacitance of CNT/Ag electrodes could be ascribed to the fact that the Ag particles could promote the contact between the electrolyte ions and the electrode surfaces, as well as the enhanced conductivity of the composite electrodes. It was also observed in Figure 3e that the addition of Ag nanoparticles significantly improved the conductivity. The experimental results showed that by doping of Ag conductive phase, the resistivity of the electrode could be 5.1 × 10^−4^ Ω/cm, which was 98.2% less than the CNT electrode. The main reason for the decrease in resistivity was the excellent conductivity of Ag nanoparticles and the good connection between Ag and CNT. It can be seen from Figure 3f that the equivalent series resistance (ESR) for the CNT/Ag-5 electrode was about 13.4 Ω/cm^2^, and CNT electrode ESR was 51.7 Ω/cm^2^. Due to the high conductivity of Ag particles, the iR dropped and was much lower than the CNT electrode. The CV curve of the CNT/Ag-5 electrode under different sweep speeds is shown in Figure 3g. The CNT/Ag-5 electrodes had very symmetrical and nearly rectangular CV curves in a potential window of 0–1 V, indicating that the device also had a excellent capacitance performance. Good linearity of GCD curves with different current densities (Figure 3h) further confirms the good electrochemical behavior of the device.

**Figure 4:**
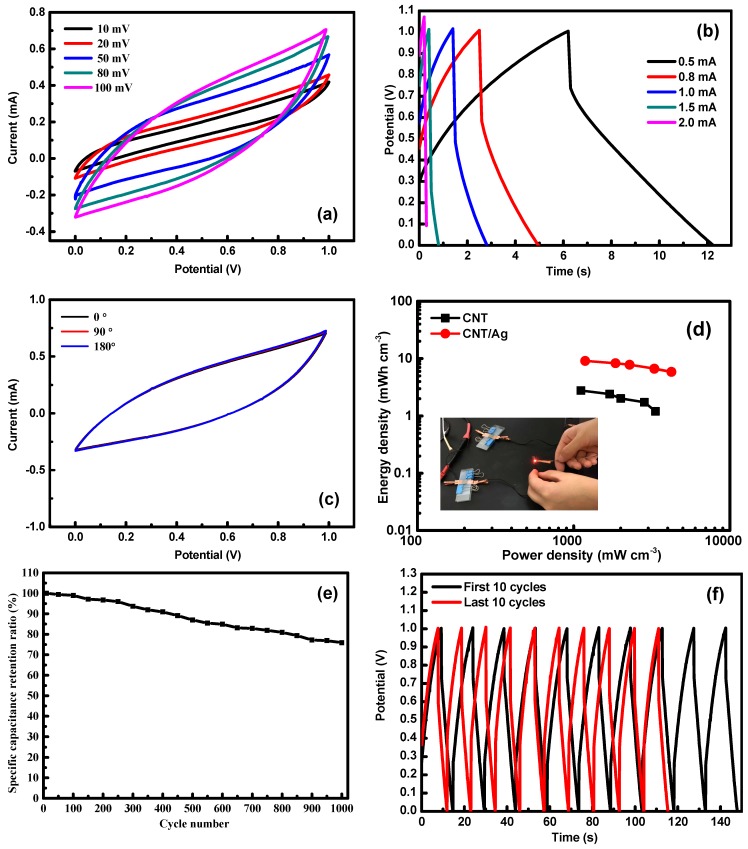
(**a**) CV and (**b**) discharge curves at different scan rates and current densities. (**c**) CV curves under different bending angles of solid-state symmetric supercapacitors. (**d**) Ragone plots of the areal energy density and power density and digital photographs of the assembled flexible solid-state symmetric supercapacitors (two in series) lighting LEDs. (**e**) Cycling stability of the flexible solid-state symmetric supercapacitors. (**f**) GCD cycling stability of the first ten cycles and last ten cycles

The CNT/Ag composite papers could be directly used as electrodes for a flexible solid-state supercapacitor (FSC) because they possess flexibility and low sheet resistance. As a proof of concept, we used two identical CNT/Ag-5 electrodes for the assembled supercapacitors, and the performance is depicted in Figure 4a. Figure 4a shows that the CV curves displayed no obvious distortion in the shape at a high scan rate of 100 mV/s. From the GCD curves (Figure 4b), we can determine that the single volumetric capacitance was 17.68 F/cm^3^ at a current density of 0.5 mA/cm^2^.

The CV curves under different bending angles of solid-state symmetric supercapacitors (at the scanning rate of 100 mV/s) is shown in Figure 4c. All of the CV curves almost overlapped under various bending conditions. This confirmed that no structure failure and capacitance loss occur upon bending up to 180°, which is the requirement for wearable applications. The specific energy and specific power were the two sticking points for evaluating the practical application in FSC.

Figure 4d presents the Ragone plot comparing the energy density and the power density of the flexible energy storage devices with the CNT/Ag-5 electrodes and CNT electrodes. It was worth noting that the assembly in this work had shown an outstanding energy density and power density; the energy density was 9.08–5.87 mWh/cm^3^ with a power density of 1.18–0.22 W/cm^3^. These powerful mechanical features allowed the device to be connected in serial geometry that provided high power to maintain a steady red LED of 1.7 V (Figure 4d). Figure 4e shows the excellent cycling stability of CNT/Ag-5 flexible solid-state symmetric supercapacitors after 1000 cycles at a current density of 0.5 mA/cm^2^. The CNT/Ag-5 sample retained up to 75.92% of its initial capacitance after the first 1000 cycles. GCD cycling stability of the first ten cycles and last ten cycles is shown in Figure 4f.

## 4. Conclusions

In summary, we reported a process for the design and fabrication of a CNT/Ag cellulose acetate membrane. The good solubility of CNT in SDBS solution, the way in which carbon nanotubes combined well with the Ag particles, and the application of the direct writing method all increased the binding force between the CNT/Ag and the cellulose acetate membrane. The incorporation of Ag into CNT largely modulated the conductivity, as well as the resulting capacitive behaviors, including the enhancement of specific capacitance, rate capability, coulombic efficiency, and cycling stability. The as-fabricated CNT/Ag-5 exhibited excellent cycling stability, with 75.92% capacitance retention. The experimental results show that by doping Ag conductive phase, the resistivity of the electrode can be 5.1 × 10^−4^ Ω/cm, which was 98.2% less than the CNT electrode. In addition, the doping of Ag nanoparticles not only improved the conductivity of the composite electrode, but also enhanced the specific volumetric capacitance up to 72.8 F/cm^3^. The as-fabricated FSC showed an outstanding flexibility for wearable applications, without showing any decline in performance under bending conditions. It also had a high energy density of 9.08–5.87 mWh/cm^3^ at a power density of 1.18–0.22 W/cm^3^.The present doping method in this work can provide more kinds of alternative electrode materials for high-performance supercapacitors. The conducting agent doping in carbon materials could be easily extended to other substances and have promising prospects in future developments.
